# Integrating chronic care with primary care activities: enriching healthcare staff knowledge and skills and improving glycemic control of a cohort of people with diabetes through the First Line Diabetes Care Project in the Philippines

**DOI:** 10.3402/gha.v7.25286

**Published:** 2014-10-21

**Authors:** Grace Marie V. Ku, Guy Kegels

**Affiliations:** Department of Public Health, Institute of Tropical Medicine, Antwerp, Belgium

**Keywords:** decision support, delivery system redesign, integration, low- and middle-income countries, primary diabetes care, self-management education and support/self-care development

## Abstract

**Background:**

This study investigated the effects of integrating primary chronic care with current healthcare activities in two local government health units (LGHU) of the Philippines on knowledge and skills of the LGHU staff and clinical outcomes for people with diabetes.

**Design:**

Integration was accomplished through health service reorganization, (re)distribution of chronic care tasks, and training of LGHU staff. Levels of the staff's pre- and post-training diabetes knowledge and of their self-assessment of diabetes care-related skills were measured. Primary diabetes care with emphasis on self-care development was provided to a cohort of people with diabetes. Glycosylated hemoglobin (HbA1c) and obesity measures were collected prior to and one year after full project implementation.

**Results:**

The training workshop improved diabetes knowledge (*p*<0.001) and self-assessed skills (*p*<0.001) of the LGHU staff. Significant reductions in HbA1c (*p*<0.001), waist–hip ratio (*p*<0.001) and waist circumference (*p*=0.011) of the cohort were noted. Although the reduction in HbA1c was somewhat greater among those whose community-based care providers showed improvement in knowledge and self-assessed skills, the difference was not statistically significant.

**Conclusions:**

Primary care for chronic conditions such as diabetes may be integrated with other healthcare activities in health services of low-to-middle-income countries such as the Philippines, utilizing pre-existing human resources for health, and may improve clinical endpoints.

Developing countries in demographic transition face problems of addressing both acute illnesses and the rising prevalence of chronic conditions (1). However, the acute disease-oriented health systems of low-to-middle-income countries (LMIC) are yet to make adjustments to accommodate this growing burden. This could be attributed to various reasons including resource constraints, absence of feasible strategies directed at chronic conditions, and difficulties in implementing chronic care strategies.

The appropriate approach to chronic care is very different from the acute disease-oriented approach practiced in most LMIC: in addition to the disease prevention and medication prescription activities usually done in acute disease care, chronic care needs to focus on disability limitation and rehabilitation (2); should give special attention to the psychosocial aspects of the patient (3); and should involve and enable the patient in taking care of the condition (4).

Various factors may influence successful implementation of the care for chronic conditions in LMIC health systems. These include human resource constraints, weak health systems, and mis-allocation of resources to suboptimally designed or implemented programs. Introducing comprehensive chronic care models *in toto*, or adding on a layer of disease-specific infrastructure to address specific chronic disease problems to existing LMIC health systems is likely to prove unfeasible and possibly detrimental.

The investigators hypothesized that integration of carefully selected elements of care for chronic conditions with other primary care activities, taking into consideration the capabilities of the health system and making use of pre-existing healthcare personnel, could be an effective approach to address the current chronic care burden in LMIC. In integrating these elements, delivery of chronic care is assimilated with the other responsibilities of the health system, is incorporated into the healthcare services and features, and is expected to be included in the patients’ experience (5).

To this end, the investigators adapted existing chronic care models (6, 7) to the context of an LMIC such as the Philippines and conceptualized a service delivery model for chronic conditions. In constructing the context-adapted chronic care model (CACCM), they considered the different elements of existing chronic care models (8, 9), methods of implementation and potential results, and the feasibility of implementing these elements to the context. The model was designed to incorporate context-adapted primary chronic care tasks with current activities of LMIC first line health systems, distributing these tasks over different pre-existing cadres of the workforce. Details of the CACCM will be discussed elsewhere.

Based on the CACCM, the investigators formulated the First Line Diabetes Care (FILDCARE) Project to integrate care for chronic conditions with other primary care activities, implementing the chronic care elements *healthcare organization*, *health service redesign*, and *decision support* on the part of the health system, and *self-management education* on the part of the person with chronic condition. Type 2 diabetes mellitus (type 2 DM) was used as the representative chronic condition. The project involved minor health service reorganization to create a chronic care team, delivery system redesign to (re)distribute certain chronic care tasks, and provision of decision support. These interventions were done to make delivery of primary diabetes care and self-management education and support (SME/S) possible without causing much additional strain to the health service. The FILDCARE Project was implemented in the local government health units (LGHU) of Batac City and Pagudpud, the Philippines. In this study, the investigators examined the effects of integrating the selected chronic care elements in the local health systems on healthcare workers’ knowledge and skills in primary diabetes care and on the glycemia of people with diabetes. The self-management education and support aspects of the project were discussed elsewhere (10).

## Background

### Models for chronic care

High-income countries have implemented models and frameworks for chronic care and chronic care delivery, most of which were derived from Wagner's Chronic Care Model (CCM) (6). The CCM was conceptualized from a primary care perspective and advocates improvements in six essential elements: self-management support, clinical information systems, delivery system redesign, decision support, healthcare organization, and community resources (8). To adapt the basic principles and elements of the CCM to something actionable in developing countries, the World Health Organization introduced the Innovative Care for Chronic Conditions framework (ICCCF). The guiding principles of the ICCCF are evidence-based decision making, population focus, prevention focus, quality focus, integration, and adaptability. It has the following essential elements for taking action: support a paradigm shift, manage the political environment, build integrated healthcare, align sectoral policies for health, use healthcare personnel more effectively, center care on the patient and family, support patients in their communities, and emphasize prevention (7).

Studies conducted on the implementation of the CCM in HIC demonstrated significant correlations between specific elements of the CCM and better health outcomes (11, 12). The number of elements of the CCM and the type and intensity of implementation may vary, depending on many contextual and organizational factors (13).

### The Philippine public first line healthcare system

Public healthcare in the Philippines was devolved in 1992. The responsibility of providing basic healthcare services for the people was handed down to local governments, specifically municipalities and cities (14). Prior to this, the country implemented a primary healthcare policy creating a large cadre of community-based health workers locally called *barangay health workers* (BHW) (15). The *barangay* (village) is the smallest unit of government; a city or a municipality is composed of several barangays. Organizationally, the BHW fall under the governance of the *barangay* and are selected to work in their respective areas of residence. They are assigned 10–20 families to attend to. Functionally, the BHW are under the LGHU. A typical LGHU is composed of at least one municipal or city health center and a number of barangay health stations, and has at least one municipal/city health officer (MHO/CHO), at least one nurse, several midwives, other paramedical and auxiliary staff, and the cadre of BHW. Currently, health services are designed in such a way that the MHO/CHO and nurse are mainly health center-based; midwives stay in the health center on some days and go to the community on specific days; and BHW are purely community-based. In general, the MHO/CHO conducts clinical consultations; the nurse carries out activities of a number of health programs (e.g. immunization); the midwives are in charge of reproductive health activities in the health center and are assigned specific villages where they carry out certain healthcare activities and directly supervise the BHW; and the BHW are responsible for dissemination of health information and health promotion activities, and perform other health-related tasks (e.g. as TB-DOTS treatment partner) for any member of the families being attended to.

The chronic condition-related activities in the LGHU are limited to informative posters on stroke, high blood pressure, diabetes, chronic lung diseases, smoking cessation, and the benefits of exercise and a healthy diet. There are also one-day annual campaigns on specific conditions as programmed by the Department of Health (16). Organized care for chronic conditions and SME/S activities is virtually non-existent and informal interviews with LGHU workers alluded to a low level of self-efficacy and proficiency in the delivery of chronic care including self-care development.

### Diabetes in the Philippines

The Philippines is predicted to be among the 10 countries worldwide with the highest numbers of people with type 2 DM by 2030 (17). Based on regular epidemiologic surveys conducted by the Philippine Food and Nutrition Research Institute, the prevalence of “new” type 2 DM as tested by a single fasting blood glucose (FBG) of ≥7.0 mmol/L increased from 3.4% in 2003 to 4.8% in 2008 together with an increase in the prevalence of known diabetes from 2.6 to 4.0% (18, 19). A rise in diabetes complications has also been noted. For renal complications alone, it is seen that 55% of Filipino diabetics will eventually develop kidney disease; in 2007 there was an increase of more than 2800 diabetic nephropathy patients requiring dialysis (20). The rapidly increasing prevalence of type 2 DM, and the poor control of disease progression and emergence of complications only show that current case management of diabetes mellitus in the Philippines is below optimum.

## Methodology

This was a prospective longitudinal two-step before–after quasi-experimental multicenter study involving a cohort of people with diabetes from two LGHU, conducted from May 2011 to February 2013. The intervention was organization of chronic care in the LGHU, consisting of minimal health service reorganization, delivery system redesign and decision support (step 1), to provide primary diabetes care emphasizing SME/S (step 2). The primary outcome of interest was change in glycosylated hemoglobin (HbA1c) of the cohort. Secondary endpoints included changes in body mass index (BMI), waist circumference (WC), and waist–hip ratio (WHR) of the cohort and changes in diabetes knowledge and care-related skills of the healthcare workers.

### Study setting

Batac [population 53,542 (21)] is a non-highly urbanized city in the island of Luzon approximately 470 km north of Metro Manila and accessible by air and land transportation. It is composed of 43 barangays and has two government health centers with barangay health stations, a tertiary-level Department of Health-operated hospital, a primary-level private hospital, a number of private multispecialty clinics and clinical laboratories, and several private drugstores/pharmacies.

Pagudpud [population 21,877 (21)], the northernmost settlement in Luzon, is a rural municipality classified to be very low in economic development. It is approximately 100 km further from Batac City. Composed of 16 barangays, it only has a basic government health unit and barangay health stations for healthcare. There are no laboratory facilities, nor any private clinics or drugstores/pharmacies.

As in many LMIC, most healthcare expenditures are out-of-pocket.

### Step 1: Decision support, minor reorganization of health services and delivery system redesign

#### Decision support: content

The investigators prepared a 32-hour training workshop on primary diabetes care and psychosocial skills development, as follows:

1. Training of skills for the provision of care and self-management education and support to the person with diabetes and the family and involvement of friends and the community for the creation of an environment aware and supportive of a healthy lifestyle and the care for diabetes, namely:
  - the biopsychosocial approach;
  - active listening;
  - patient empowerment;
  - family empowerment; and
  - social mobilization.

based on family medicine principles, which comprised Module 1.

1. Education and training of necessary knowledge and skills for primary diabetes care that includes:
  - basic pathophysiology of diabetes and current Philippine clinical practice guidelines on the diagnosis and management of diabetes mellitus (Module 2);
  - lecture, demonstration and hands-on training on anthropometric measurements (weight, height, waist and hip circumference) and computations (BMI, waist–hip ratio), blood pressure determination, capillary blood glucose testing, and interpretation of these anthropometric and clinical parameters following international guidelines and standards (Module 3);
  - lecture on foot care and foot care advice and a workshop on foot examination based on international standards for foot care (Module 4);
  - lecture on the diabetic diet, food exchanges and glycemic indices and a workshop on dietary counseling (Module 5);
  - lecture and two-way demonstrations on physical activity and exercise based on international guidelines on physical activity and exercise (Module 6).

The first module was added in order to train the healthcare workers on incorporating psychosocial approaches into the usual (purely) biomedical approach to acute conditions that is currently being practiced. In order to pave the way for (full) self-management of the person with diabetes, moves should be made to veer away from the provider-directed, compliance-oriented approaches that are typically practiced in the care for acute conditions; rather a collaborative approach directed toward patient empowerment (22) and patient engagement (23) is needed. It has been shown that healthcare providers can support patients’ self-care efforts through more effective communication (24). To better recognize real concerns of the patient, this patient–provider communication needs to use the biopsychosocial approach and active listening (25). Finally, the healthcare system would be mobilized toward promoting and improving self-care.

The principal investigator conducted the lectures while the workshops were facilitated by the principal investigator and two registered nurses who were trained prior to the workshop. With the exception of Module 2 (basic pathophysiology and Philippine clinical practice guidelines for the diagnosis and case management of DM type 2), which consisted only of a lecture, all modules were composed of introductory lectures, two-way demonstrations, and hands-on workshops.

The trainers provided written copies of the lectures and other course materials to the participants. The investigators prepared the modules in accordance with current standards and practices for primary diabetes care and self-care development (26–31), but adapted to the local context and the capabilities of the healthcare services and healthcare workers.

#### Decision support: participants

The training workshop was separately conducted in Batac City and in Pagudpud. Attendees were the respective CHO and MHO, LGHU nurses, midwives, and BHW. Basic diabetes knowledge was tested and self-assessment of primary diabetes care-related skills was gathered from these healthcare workers before and after the training. The basic diabetes knowledge test is composed of 24 questions based on the Fitzgerald et al. Diabetes Knowledge Test (32) and the Garcia et al. Diabetes Knowledge Questionnaire (33). The self-assessment covered the different skills taught in the workshop divided into three categories: 1) the biopsychosocial approach and active listening skills; 2) anthropometric measurements, computations, and interpretations; and 3) clinical measurements and interpretations, and non-pharmacologic approaches. The self-assessment made use of a 5-point Likert scale with 1 being the lowest (“don't know”) and 5 being the highest (“can teach about it”) possible ratings. Written informed consent was obtained prior to the knowledge test and self-assessment.

#### Minor reorganization and delivery system redesign

**Fig. 1:**
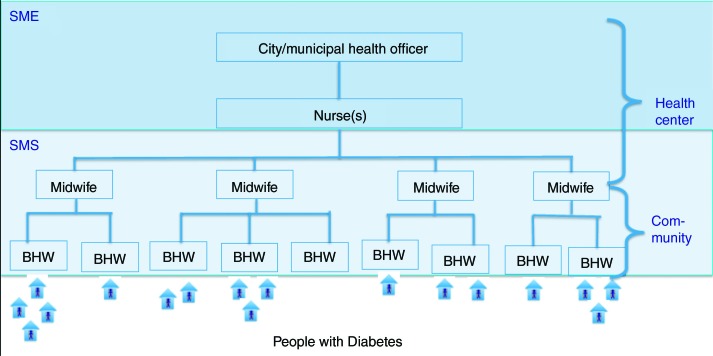
Self-management education and support strategy utilizing pre-existing local government unit healthcare workers in their present organization.

The chronic care team, composed of the MHO/CHO, nurse, midwives, and BHW, was created. The organization of the healthcare workers was retained and specific tasks relative to the delivery of primary diabetes care and SMES were distributed. Clinical consultations were retained as a task of the MHO/CHO. Self-management education (SME) activities were assigned to either the MHO/CHO or the nurse. Self-management support (SMS) activities were re-assigned to the midwives and BHW (Figure 1).

### Step 2: Primary diabetes care and SME/S project implementation

After the training, the healthcare workers were requested to enroll people with diabetes from their localities in the FILDCARE Project. The principal investigator or the trained field researchers provided full project information and obtained written informed consent from each of the study participants. HbA1c was measured making use of *HbA1cNow* (Bayer HealthCare, Makati City, Philippines) before implementation and one year after full implementation of the project. The *HbA1cNow* is a point-of-care test that conforms to the National Glycohemoglobin Standardization Program protocol. The researchers also collected pre- and post-implementation data for the BMI, WC, and WHR.

#### Inclusion/exclusion criteria

Criteria for inclusion in the FILDCARE Project were: diagnosis of type 2 diabetes, age ≥20 years, and willingness to participate in the project. The trained healthcare workers provided primary diabetes care and DSME/S to the project participants. Data gathered from the project participants were further screened for inclusion in statistical analysis. Inclusion criteria for analysis were: completeness of interview data, pre- and post-implementation HbA1c values and pre- and post-implementation anthropometric measurements. Exclusion criteria were: pregnancy and a positive medical history of anemia (sickle cell, iron deficiency) and end-stage renal disease.

#### FILDCARE Project SME/S strategy

The SME/S strategy was formulated based on a conceptual framework constructed by the investigators (34), where various external and internal influences may affect the perceived self-efficacy of the person with diabetes and motivate or demotivate adoption of and adherence to self-care. One-on-one diabetes self-management education (DSME) was started either by the city/municipal health officer or the LGHU nurse, initially assisted by the principal investigator and/or the FILDCARE Project nurse, during consultations at the government health unit. Consultations and the concomitant DSME sessions were done at least once every three months. DSME was conducted in a conversational and interactive manner, embedded in the clinical consultation, thus transforming the purely biomedical character of the encounter between the physician or the nurse and the person with diabetes into a biopsychosocial one. Duration of the initial DSME session ranged from 20 to 30 minutes and the succeeding sessions from 5 to 15 minutes. Project-provided written materials on healthy eating, exercise, and glycemic goals were given out during the sessions. Community-based diabetes self-management support (DSMS) was continued by the BHW and, on occasion, the midwives. DSMS was provided informally through home visits, either purposively or opportunistically when the BHW performs other health activities to (other) members of the household. This way, the DSMS sessions would at times include the family, providing chances for family meetings and healthy lifestyle promotion to family members. DSMS sessions were also conducted in the barangay health stations where the BHW and midwives would be found on specific days two to four times a month. DSMS was provided at least once a month. The frequency and duration of DSME/S depended primarily on the demand of the person with diabetes. The DSME/S approach was collaborative and interactive rather than rigidly structured. After the opening DSME where the different aspects for self-management were discussed, the opinion and choices of the person with diabetes on the topics to be tackled in succeeding DSME/S sessions were considered. Active listening skills (introduced in the decision support training workshop) were employed where the person with diabetes is encouraged to talk about the condition and caring for the condition – perceptions, problems and difficulties encountered, accomplishments, etc. – which were then addressed in an interactive manner, usually ending up with an action plan or reinforcement of a previous action plan.

### Definitions

Improved knowledge and self-assessment of skills of the healthcare workers were defined as a positive increase in the respective ratings in the post-test.

The investigators used HbA1c level of <7% as the cut-off for good glycemic control (31).

BMI and WC were classified based on suggested Asian cut-off values (35) and WHR risk categorization was based on World Health Organization (36) recommendations.

### Statistics

Statistical analyses were done making use of the statistical package Stata/IC (37) version 11.0. Wilcoxon signed-rank test was used to compare the pre- and post-training results of the healthcare workers. Wilcoxon signed-rank test was used to compare the pre- and post-implementation results of the HbA1c and secondary clinical endpoints of the FILDCARE Project participants. Test of proportions was used to compare the proportions of project participants who achieved glycemic and obesity/adiposity goals prior to and after program implementation. Mann-Whitney test was used to compare the changes in knowledge and skills of the healthcare workers and the changes in HbA1c among people with diabetes whose community-based care providers’ knowledge and skills improved against those whose community-based care providers’ knowledge and skills did not. Cronbach's alpha was used to test for internal consistency of the questionnaires used.

### Ethical considerations

This research was approved by the Institutional Review Boards/Ethics Committees of the University of Antwerp and Institute of Tropical Medicine Antwerp, Belgium (Belgian Reg. No. B30020109490); and the Mariano Marcos Memorial Hospital and Medical Center, Batac City, the Philippines. It was conducted with permission from the government of the Province of Ilocos Norte and the Ilocos Norte Provincial Health Office; the government of the City of Batac and its City Health Office; and the government of the Municipality of Pagudpud and its Municipal Health Office.

## Results

### Training of healthcare workers

Complete pre- and post-training diabetes knowledge tests and self-assessments were collected from 110 of the 125 healthcare workers who attended the workshop. Table 1 lists the positions and levels of education of the healthcare workers who took the knowledge and self-assessment pre- and post-training tests. It is interesting to note that 80% (70/87) of the BHW had at least some secondary school education and almost 30% (24/87) have a college degree. Evaluation of the workshop's results on the healthcare workers (Table 2) showed a significant increase in correct answers to the knowledge test (*p*<0.001); and significant increase in self-assessment of biopsychosocial and communication skills (*p*<0.001), anthropometric measurement and interpretation skills (*p*<0.001), and skills on basic clinical and non-pharmacological approaches to diabetes (*p*<0.001) in both sites. The improvement in knowledge was more marked among the BHW, whose post-test knowledge ratings increased by an average of 25.0%, as compared to the formal healthcare workers whose post-test knowledge ratings increased by an average of 12.5% (*p*=0.013). There were no significant differences in the change in knowledge among healthcare workers from Batac City as compared with healthcare workers from Pagudpud, but the healthcare workers from Pagudpud gave more optimistic self-assessment of improved skills. The changes in the self-assessed skills were likewise higher among the BHW. Knowledge improved in 91.8% of the participants; assessment of biopsychosocial and communication skills improved in 94.5%, anthropometric measurements and interpretation skills in 90.9%, and clinical and non-pharmacologic approaches in 91.8%. Results of Cronbach's alpha analysis are also listed in Table 2.

**Table 1:** Positions and levels of education of healthcare workers who participated in the training with complete pre- and post-test and self-assessments

	All, *n*=110	Batac City, *n*=67	Pagudpud, *n*=43
Position			
Health officer	1 (0.9%)	0	1 (2.3%)
Nurse	6 (5.5%)	5 (7.5%)	1 (2.3%)
Midwife	16 (14.5%)	10 (14.9%)	6 (14%)
Barangay health worker	87 (79.1%)	52 (77.6%)	35 (81.4%)
Education			
Doctor of medicine	1 (0.9%)	0	1 (2.3%)
College degree	46 (41.8%)	29 (43.3%)	17 (39.5%)
College level	6 (5.5%)	6 (9%)	0
>6–10 years of school	46 (41.8%)	29 (43.3%)	17 (38.5%)
≤6 years of school	9 (8.2%)	3 (4.5%)	6 (14%)
Not indicated	2 (1.8%)	0	2 (4.7%)

In the Philippines, health officers are physicians and have a doctor of medicine degree; nurses and midwives are graduates of their respective college degrees (Bachelor of Science in Nursing and Bachelor of Science in Midwifery). All three cadres of healthcare workers need to be licensed by the Philippine Professional Regulations Commission before they can practice their professions.

**Table 2:** Changes in knowledge and self-assessment of skills of the healthcare workers, classified into formal healthcare workers (health officer, nurses, midwives) and the BHW

		All (*n*=110)		Formal healthcare workers (*n*=23)		BHW (*n*=87)		
			
		Median (binomial interpolation of confidence intervals)	Wilcoxon signed-rank test *p* value	Median (binomial interpolation of confidence intervals)	Wilcoxon signed-rank test *p* value	Median (binomial interpolation of confidence intervals)	Wilcoxon signed-rank test *p* value	Cronbach's alpha
Diabetes knowledge test,% correct answers	Pre-test	54.2 (50.0–58.3)	<0.001	70.8 (66.7–75.0)	<0.001	45.8 (41.7–54.2)	<0.001	0.903
	Post-test	75.0 (70.8–78.3)		87.5 (79.2–87.8)		70.8 (66.7–75.0)		
Self-assessment, biopsychosocial approach domain*	Pre-test	1.32 (1.11–1.73)	<0.001	2.64 (1.99–3.46)	<0.001	1.09 (1.00–1.27)	<0.001	0.972
	Post-test	3.41 (3.20–3.70)		3.91 (3.64–4.0)		3.18 (3.00–3.40)		
Self-assessment, anthropometrics*	Pre-test	1.40 (1.22–1.60)	<0.001	2.60 (1.77–3.51)	0.001	1.40 (1.01–1.49)	<0.001	0.972
	Post-test	3.04 (3.00–4.00)		4.00 (3.00–4.9)		3.00 (3.00–4.00)		
Self-assessment, clinical and non-pharmacological skills*	Pre-test	1.32 (1.21–1.64)	<0.001	2.93 (2.14–3.29)	<0.001	1.29 (1.14–1.43)	<0.001	0.967
	Post-test	3.5 (3.30–3.84)		3.50 (3.36–5.00)		3.5 (3.14–3.85)		

* Score on a 1–5 Likert scale.

### Primary diabetes care and self-care development

A total of 203 people with diabetes were enrolled to the FILDCARE Project, 134 in Batac City and 69 in Pagudpud. Complete pre- and post-implementation data were gathered from 164 (80.8%) FILDCARE Project participants. In Batac City, complete data were collected from 108 participants (80.6% of Batac City participants). Of the 26 with incomplete data, four died, four migrated, and 18 refused any further HbA1c testing. In Pagudpud, complete data were collected from 56 participants (81.2%); five refused any HbA1c testing from the outset, two refused further testing after the baseline, two refused post-implementation interview and four migrated. None were found to have any of the listed exclusion criteria. Project participants with complete pre- and post-implementation data were included in the analyses. Based on the pre- and post-implementation HbA1c results and an alpha of 0.05, the computed power of this study involving 164 people with diabetes is 99.95%.

Demographic data of the participants are listed in Table 3.

**Table 3:** Demographics of people enrolled in the FILDCARE Project

		All, *n*=164	Batac, *n*=108	Pagudpud, *n*=56

Male gender, *n* (%)		42 (25.6%)	30 (27.8%)	12 (21.4%)
Age in years	Average	57.1	57.8	55.8
	Median	57	59	55
	Range	27–83	33–83	27–79
Number of years with diabetes	Average	4.5	5.4	2.8
	Median	2	3	1
	Range	0.5–28	0.5–28	0.5–16
Level of education	0–6 years	43 (26.2%)	22 (20.4%)	21 (37.5%)
	7–10 years	63 (38.4%)	43 (39.8%)	20 (35.7%)
	>10 years	58 (35.4%)	43 (39.8%)	15 (26.8%)

Table 4 summarizes the changes in pre- and post-implementation clinical endpoints of the FILDCARE Project participants. The changes in HbA1c ranged from −7.1% to +5.3%, with an average change of −0.49% (95% CI=−0.78 to −0.19). Regardless of level of control, HbA1c decreased in 99 (60.4%) of the project participants with an average reduction of −1.44 HbA1c percentage points and an absolute reduction of −1.0 HbA1c percentage points or more in 26.2%. HbA1c remained the same in 13 (7.9%) and increased in 52 (31.7%).

**Table 4:** Pre-implementation and post-implementation values of the clinical endpoints of the FILDCARE Project participants

	ALL, *n*=164			BATAC, *n*=108			PAGUDPUD, *n*=56		
		
	Pre-implementation	Post-implementation		Pre-implementation	Post-implementation		Pre-implementation	Post-implementation	
								
	Clinical endpoints	Median (binomial interpolation of confidence intervals; range)		*p* Value (Wilcoxon signed-rank test)	Median (binomial interpolation of confidence intervals; range)		*p* Value (Wilcoxon signed-rank test)	Median (binomial interpolation of confidence intervals; range)		*p* Value (Wilcoxon signed-rank test)
Glycosylated hemoglobin, %	7.7 (7.2–8.2; 5.2–>13.0)	6.9 (6.8–7.5; 4.7–>13.0)	<0.001	7.8 (7.06–8.8; 5.2–>13.0)	7.1 (6.8–8.0; 4.8–>13.0)	0.0143	7.4 (6.7–8.2; 5.3–>13.0)	6.8 (6.5–7.7; 4.7–>13.0)	0.0185
Body mass index, in kg/m^2^	23.7 (23.4–24.5; 15.7–39.5)	23.2 (22.9–24.1; 15.0–35.2)	0.059	23.8 (23.4–24.3; 16.0–33.6)	23.2 (22.2–23.9; 16.7–33.2)	0.0139	23.0 (22.2–24.7; 15.8–39.5)	23.4 (22.2–25.0; 15.0–35.2)	0.698
Waist circumference, in cm	85.0 (83.8–86.4; 68.5–112.0)	83.0 (82.0–85.0; 63.0–108.0)	0.011	85.0 (83.8–88.0; 60.0–112.0)	83.0 (82.0–86.0; 63.0–108.0)	<0.001	84.5 (82.2–86.4; 70.0–107.0)	84.2 (81.2–86.0; 69.0–107.5)	0.701
Waist-hip ratio	0.90 (0.89–0.91; 0.77–1.56)	0.89 (0.88–0.89; 0.65–1.15)	<0.001	0.90 (0.89–0.91; 0.77–1.1)	0.89 (0.88–0.90; 0.75–1.1)	0.006	0.91 (0.89–0.92; 0.81–1.56)	0.88 (0.87–0.90; 0.65–1.0)	0.006

Table 5 categorizes the pre- and post-implementation clinical endpoints of the project participants according to defined norms. The proportion of FILDCARE Project participants with good glycemic control significantly increased for both sites (*p* values: All=0.014; Batac<0.001; Pagudpud=0.034). The noted decrease in WC (−1.4 cm) and WHR (−0.2) were statistically significant (*p*=0.011 and <0.001, respectively). The increase in the proportion of project participants classified to have normal BMI (from 36.0 to 39.6%), normal WC (from 32.3 to 37.2%) and categorized as low risk on the basis of WHR (from 18.9 to 25%) were not statistically significant (*p* values of 0.494, 0.354 and 0.286, respectively).

**Table 5:** Pre- and post-implementation categorization of the FILDCARE Project participants according to set criteria of the clinical endpoints

			Proportions, *n* (%)		

Clinical endpoints		Reference values	Pre-implementation	Post-implementation	*p* Value (test of proportions)
Glycosylated hemoglobin (%)					
Good glycemic control	All	<7.0%	61 (37.2%)	83 (50.6%)	0.014
	Batac		38 (35.2%)	54 (50.0%)	<0.001
	Pagudpud		23 (41.1%)	29 (51.8%)	0.034
Body mass index classification (kg/m^2^)					
Underweight		<18.5	6 (3.7%)	11 (6.7%)	0.213
Normal		18.5–22.9	59 (36.0%)	65 (39.6%)	0.494
Overweight		23–24.9	45 (27.4%)	35 (21.3%)	0.199
Obese type 1		25–29.9	47 (28.7%)	46 (28.1%)	0.902
Obese type 2		≥30	7 (4.3%)	7 (4.3%)	1.000
Waist circumference risk stratification (cm)					
Low risk		Male <90 cm Female <80 cm	53 (32.3%)	61 (37.2%)	0.354
Waist-hip ratio risk stratification					
Low risk		Male ≤0.95 Female ≤0.80		40 (24.4%)	0.286
Moderate risk		Male=0.96–1.0 Female=0.81–0.85	31 (18.9%)	33 (20.1%)	0.781
High risk		Male >1.0 Female >0.85	102 (62.2%)	91 (55.5%)	0.217

A total of 21 (12.8%) participants (Batac=10, Pagudpud=11) were under the care of the BHW whose knowledge and self-assessment of skills did not improve. The average reduction in HbA1c among these 21 participants (−0.40 HbA1c percentage points) was somewhat less than but not statistically different from the change in HbA1c among the rest of the participants (−0.50 HbA1c percentage points; *p*=0.553).

## Discussion

Health systems adopting models for chronic care are expected to improve their performance (38, 39). Examples in LMIC include the CCM-based Vera-Cruz Initiative for Diabetes Awareness in Mexico, which reported improved glycemia 18 months after implementation (40). In Rwanda, the Integrated Care for Chronic Conditions Model was used as a roadmap in designing a system of care for people with HIV–AIDS, with excellent results (41, 42). However, context-adaptations of models for chronic care with the aim of integrating care for chronic conditions with current healthcare activities in LMIC are still rare. Based on past policies, it seems that these LMIC would envisage implementation of these chronic care models – if any – as additional vertical programs that need to be introduced to health systems as separate packages with exclusively dedicated resources. In contrast, in the FILDCARE Project, we hypothesized that it is possible to apply basic CCM principles by embedding – or integrating – selected elements in existing primary care structures and activities, with positive results.

Although a number of aspects of chronic care ideally require expertise, skill, and specialized personnel that LMIC may not have the capacity to supply, there are certain chronic care activities that can be translated to low-resource settings, a portion of which may be shifted to non-medical professionals. Experts have recommended redistribution of chronic care tasks to address scarcity of human resources for health (43). Such could also be resorted to in order to decrease the burden on LMIC professional healthcare workers who have to deliver care for both acute and chronic conditions. The utilization of non-medical people (e.g. community health workers and people with similar chronic conditions), particularly in self-care development, has been investigated in the past and has produced favorable results (44, 45). For the FILDCARE Project, the investigators examined standards of diabetes care and dissected different SME/S programs and curricula (22, 23, 28–31, 46–48) to explore content and structure that have the potential to be utilized and incorporated in the Philippines; classified these primary diabetes care and SME/S activities according to standardizability and the proficiency level required and redistributed these activities to the pre-existing healthcare staff according to their levels of expertise; and trained the LGHU staff accordingly. Additionally, the healthcare workers were trained on the biopsychosocial approach and active listening to facilitate a more active involvement of the person with diabetes towards self-care. This way the organization of chronic care providers and (the usual) chronic care delivery design were adjusted to the context, and decision support was implemented.

The training workshop pre-test and initial self-assessment of the healthcare workers showed low knowledge of and skills in basic primary diabetes care and psychosocial approaches, which significantly improved after the training. The BHW showed greater confidence in their self-assessment of diabetes care-related and psychosocial skills after the training. More importantly, activities appurtenant to the project seem to be associated with significantly improved glycemic control of the project participants, whether in the LGHU of Batac or that of Pagudpud. After implementation of the FILDCARE Project, HbA1c of the participants decreased by an average of 0.49 HbA1c percentage points (the median value was 0.8) and the number of people with diabetes in good glycemic control increased by 36.1%. Post-implementation HbA1c levels decreased in 60.4% of the participants. This reduction in HbA1c regardless of glycemic control is likely to equate to decreased risks of developing diabetes-related complications in the individual (30, 49). Moreover, even if very few of the project participants achieved goals for adiposity/obesity, the significant decrease in median WC and WHR measurements of the participants remains.

The statistically negligible effects on HbA1c changes of people under the care of the BHW whose knowledge and self-assessment of skills improved over those who did not improve may be attributed to the fact that SME/S is delivered by a team composed of the CHO/MHO, nurse, midwife, and BHW; people with diabetes seen by the BHW also have SMS sessions with the midwife and SME sessions with the CHO/MHO and nurse. However, this lesser reduction in HbA1c of the people under the care of the BHW whose knowledge and self-assessment of skills did not improve may need to be explored further.

The equally significant improvements in HbA1c in the urban LGU of Batac City where resources for health and healthcare are more readily available and in the rural LGU of Pagudpud where resource constraints are more marked and access to medicine and laboratory tests are more problematic suggests that interventions which offer the flexibility to be adjusted – the context-adaptation of the CCM on the part of the health system and the use of the biopsychosocial approach coupled with active listening on the part of self-care development of the individual person with chronic condition – can work in different situations.

These interventions were implemented making use of pre-existing staff of the local health systems, integrating chronic care activities with other healthcare activities. The inclusion of SME during clinical consultations of people with diabetes and the addition of SMS during the regular visits of the midwives in their villages of responsibility and of the BHW to the homes of families under their care did not pre-empt other activities and services. Acceptance of the FILDCARE Project among the healthcare workers was facilitated with the decision support that was provided which, at the least, bolstered their perceived self-efficacy to deliver primary diabetes care, and with the redistribution of some chronic care activities which addressed task issues.

### Study limitations

The absence of a control group limits the findings of this study. Other factors may also have contributed to the significant improvement in glycemic control of the project participants beyond the organization of primary diabetes care and SME/S strategy implemented. These may include sensitization of the involved communities including local government officials to chronic conditions such as diabetes; and the access to HbA1c test and immediately available results upon consultation at the health unit (50). Furthermore the effects of accessibility of diabetes drugs, specialists, and diabetes care-related equipment were not evaluated. Changes in other measured endpoints and the project participants’ knowledge, attitudes, and perceptions will be discussed elsewhere. Some bias may have occurred in the assessment of the healthcare workers as not all workshop participants participated in both the pre- and post-tests, but such an effect can be considered negligible.

### Way forward

The project has garnered strong local government support, which has led to local policies on community promotion activities for the prevention of lifestyle-related chronic conditions. The investigators left training and other materials with the CHO/MHO for future use. The healthcare workers, with the encouragement and support of local government officials, have continued to conduct the SME/S activities even after the conclusion of the project. Beyond these, a public-private partnership is being considered to improve access to blood glucose testing by introducing low-cost point-of-care blood glucose monitors to the LGHU. In Batac City, regular community-based screening for diabetes has been ongoing since 2012; there are thrice-weekly LGHU-guided and led aerobic exercise sessions in the City Hall since 2013; and the Batac City Health Officer has taken initiatives to expand access to diabetes medicine. Certain free oral hypoglycemic agents and anti-hypertensive medications are now available.

## Conclusion

The FILDCARE Project incorporated chronic care with current healthcare activities making use of pre-existing human resources for health. In providing the needed training to increase knowledge on diabetes and to develop the necessary communication and primary diabetes care-related skills, stronger decision capacity was built among the healthcare workers. Minimal reorganization of the health service and delivery system redesign were done. Although the design of the FILDCARE Project may be far-removed from those seen in HIC, basic elements of chronic care and SME/S were successfully taught to the healthcare workers and incorporated in the FILDCARE Project. The favorable results of this study in both the urban and the rural sites strongly suggest that care for chronic conditions may be introduced and/or improved through low-resource, context-adapted, simplified strategies. The increase in knowledge and skills of the healthcare workers during their training, and the changes in HbA1c and in the proportion of people in good control of their diabetes are significant and clinically relevant. Finally, the observation of a decrease in HbA1c in the majority of the participants is encouraging in view of the fact that glycemic control is not known to improve spontaneously through time.

### Implications for policy and further research

The FILDCARE Project showed that chronic care may be integrated in current healthcare activities where organized care for chronic conditions was previously non-existent. A 32-hour basic training workshop on knowledge and skills of the healthcare workers can be translated to better diabetes care in a resource-constrained routine setting. The positive outcomes were achieved without entailing much additional demand on the health system. Pre-existing human resources can be utilized and standardizable tasks that do not require much expertise can be shifted to a less qualified workforce. In this case, the investigators took advantage of the large and well-established cadre of BHW to deliver SMS, thereby decreasing part of the additional burden of chronic care that may be imposed on the professional healthcare workers who are already weighted down with acute care delivery. The familiarity of the BHW with the people under their care may have contributed significantly to address sociocultural barriers to adequate self-care and may have positively contributed to the dynamics of the program. Such issues and similar questions on the more precise mechanisms this program triggered to produce its results will have to be further investigated. It is likely that continuous capacity building to the healthcare teams through refresher courses or supportive supervision will have to be provided to ensure long-term benefits from initiatives like the FILDCARE Project. This sustainability issue also will require further investigation.

LMIC facing problems with chronic conditions and the care for chronic conditions may take inspiration from this experience to initiate integration of CCM elements that fit their context, as was done in this project.
